# Supplementary material for: Gene-Level, but Not Chromosome-Wide, Divergence between a Very Young House Fly Proto-Y Chromosome and Its Homologous Proto-X Chromosome
Source: Mol Biol Evol. 2020 Sep 28;38(2):606–18. doi: 10.1093/molbev/msaa250 (PMC7826193; doi:10.1093/molbev/msaa250)
Supplement: msaa250_Supplementary_Data [file msaa250_supplementary_data.zip › msaa250-suppl_data/R2_supplementary_materials.pdf]

Supplementary Materials

Identification of two candidate proto-X/proto-Y loci

We identified one contig containing *Mdmd* in the III^M assembly (ctg2382) that contains the same three genes as another contig (ctg1607) without *Mdmd* (Figure 2A). We infer the contig with *Mdmd* to be the proto-Y sequence, and the one without to be from the proto-X. There are two additional contigs containing *Mdmd* in our III^M genome assembly, but we did not find any corresponding proto-X contigs for these (Supplementary Figure 1). These other two contigs with *Mdmd* either contain loci without sufficient X-Y divergence to assemble into separate proto-X and proto-Y contigs, or we did not sequence deep enough to capture enough proto-X reads to assemble the proto-X gametolog. The male-determining regions of the house fly proto-Y chromosomes contain both complete and truncated copies of *Mdmd* (Sharma et al. 2017), and ctg2382 contains two truncated copies of *Mdmd*. The genomic region corresponding to this contig is found in a single scaffold of the reference genome (NW_004774683.1), which was previously assigned to the third chromosome (Meisel and Scott 2018). There are three genes at this locus, and they are present in the same order in the reference genome, the proto-Y contig, and proto-X contig (Figure 2A).

We found the second locus using a *k*-mer comparison approach to test for contigs containing sequences that are enriched in males relative to females (Carvalho and Clark 2013). Here, we compared *k*-mers in our Nanopore III^M male reads and the Illumina reads used to generate the reference (female) genome assembly (Scott et al. 2014) to our III^M male assembly. We then calculated the percent of each contig in our III^M assembly that is unmatched by female reads (%UFR), which we expect to be 100% for a Y chromosome contig that only contains male-specific *k*-mers. We did not identify any contigs with greater than 27.4%UFR (Supplementary Figure 1), which is consistent with the minimal sequence divergence between the proto-Y and proto-X chromosomes (Meisel et al. 2017). To determine an expectation for what constitutes a %UFR of a proto-Y contig in our assembly, we calculated %UFR for the three contigs that each contain copies of *Mdmd* (Supplementary Figure 1). The largest %UFR of any contig with

Mdmd is 13.8% (ctg3705). We used this value as a threshold to find proto-Y contigs that do not contain *Mdmd*.

We found 275 contigs with a %UFR>13.8, nine of which (Supplementary Table 1) contain protein-coding genes that were previously assigned to the third chromosome (Meisel and Scott 2018). One gene (LOC101898200, which encodes histone H3) was found on six different contigs with %UFR>13.8 (and an additional 27 contigs with %UFR<13.8). Histone gene clusters are a common feature of most metazoan genomes, and the multiple copies are under tight regulatory control (McKay et al. 2015; Duronio and Marzluff 2017).These contigs with high %UFR and copies of histone H3 genes are either false positives or not of substantial phenotypic relevance. Two of the other contigs with %UFR>13.8 (ctg3539 and ctg8407) each contain 1-2 genes that are found as multiple copies on other contigs (all with a %UFR<13.8). One of those genes (LOC109612838 on ctg3539) encodes a protein with predicted reverse transcriptase and RNase H activity, suggesting that it is a retrotransposon (Finnegan 2012). Another gene (LOC109613819 on ctg8407) encodes a protein with zinc finger and integrase domains, also consistent with a transposable element (Volff 2006). We exclude these contigs from our subsequent analysis because they likely reflect transposable element expansions within the house fly genome. We only found one contig with a %UFR>13.8 (ctg2522, which has 16.9%UFR) that has a single corresponding contig (ctg1519) with the same four genes (Supplementary Table 1). We assigned ctg2522 to the proto-Y chromosome and ctg1519 to the proto-X (Figure 2B). All four genes shared by both contigs are mapped to the third chromosome in the reference genome. These four genes are present in the same order and same orientation in the reference genome (on scaffold NW_004764689.1), the proto-Y contig, and proto-X contig.

DNA isolation with phenol/chloroform protocol

A single genotypic male and a single sex-reversed male with detached wings were each transferred to a 1.5 mL tube with 0.5 mL homogenization buffer (4.1 g sucrose, 15 mL 1M Tris-HCl pH 8.0, 0.5M EDTA, 100 mL dH_2O) and then homogenized using a pestle set into a tissue grinder homogenizer. To each tube we added 40 uL of 10% SDS and 2.5 uL of 10 mg/mL Proteinase K, and then we incubated the tube at 65°C for 30 min. We next added 2 uL of 4

mg/mL RNase to each tube and incubated at 37°C for 15 min. We added 48 uL of 5M KAc to each tube and placed on ice for 30 min. Then we centrifugated the tubes at 14000 rpm for 10 min at 4°C, and the supernatant was transferred into a new tube using a wide-bore pipette tip (all subsequent steps of transfer and mixing during DNA extraction were also done with wide-bore pipette tips to prevent DNA shearing). We added 250 uL phenol and 250 uL chloroform to the extracted supernatant in the new tube, mixed briefly, spun at 14000 rpm for 15 min at 4°C, and then transferred the supernatant into a new tube. We next added 500 uL chloroform to the supernatant in the new tube, mixed well, and spun a 14000 rpm for 5 min at 4°C. We then transferred the supernatant into a new tube. We added 40 uL of 3M NaAc and 800 uL of 95% ethanol to the supernatant in the new tube, mixed briefly, spun at 14000 rpm for 15 min at 4°C, and then carefully poured off all supernatant. We next added 800 uL of 70% ethanol to the remaining pellet, mixed briefly to wash the pellet, spun at 14000 rpm for 15 min at 4°C, removed the supernatant, and then resuspended the pellet in 30 uL of nuclease-free water.

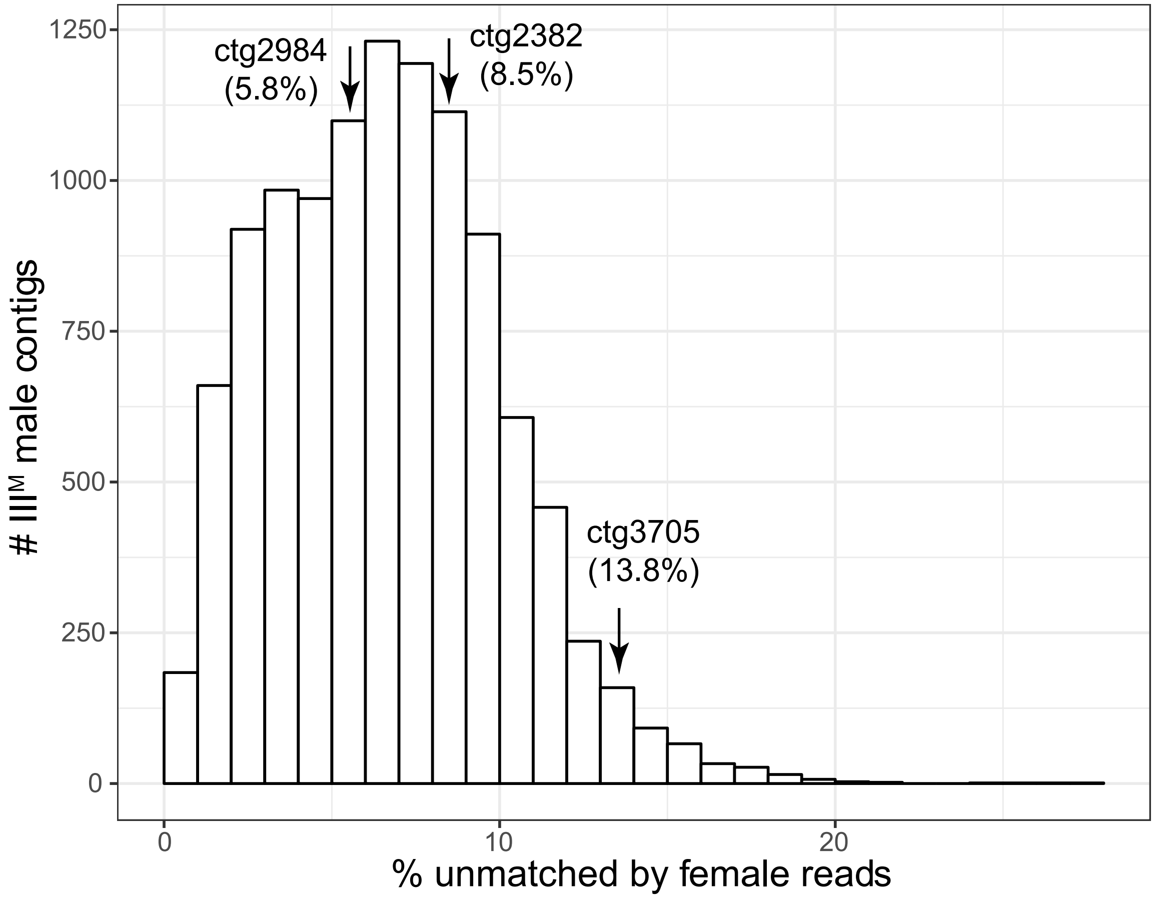

Supplementary Figure 1. Histograms of the percent unmatched by female reads (%UFR) for all contigs in the III^{M} male genome assembly. Three contigs with *Mdmd* are shown, with their %UFR in parentheses.

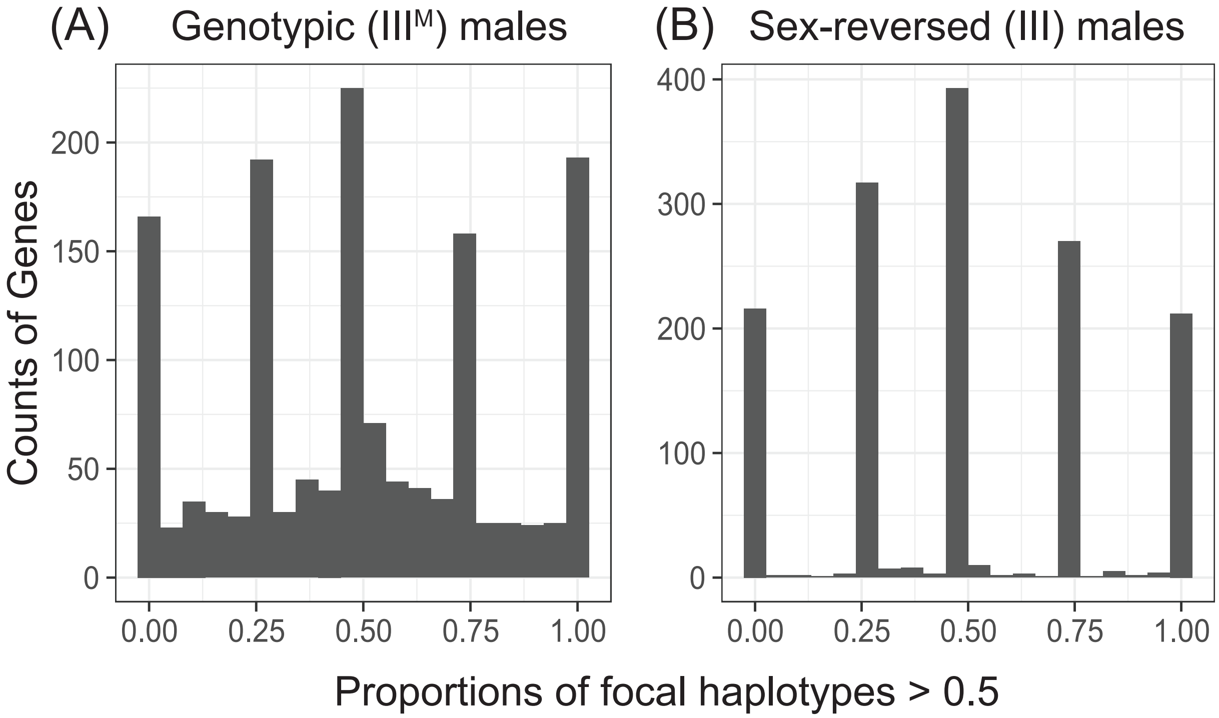

Supplementary Figure 2. Histograms of third chromosome genes in different ASE categories. ASE was measured in (A) genotypic (III^M/III) and (B) sex-reversed (III/III) males. ASE is measured as the proportion of iterations in which a focal haplotype is >0.5 of the alleles expressed across iterations in an MCMC simulation. We consider a gene to have ASE if the proportion is <0.125 or >0.875. Genes with a proportion of focal haplotypes between 0.375 and 0.625 are classified as not having ASE.

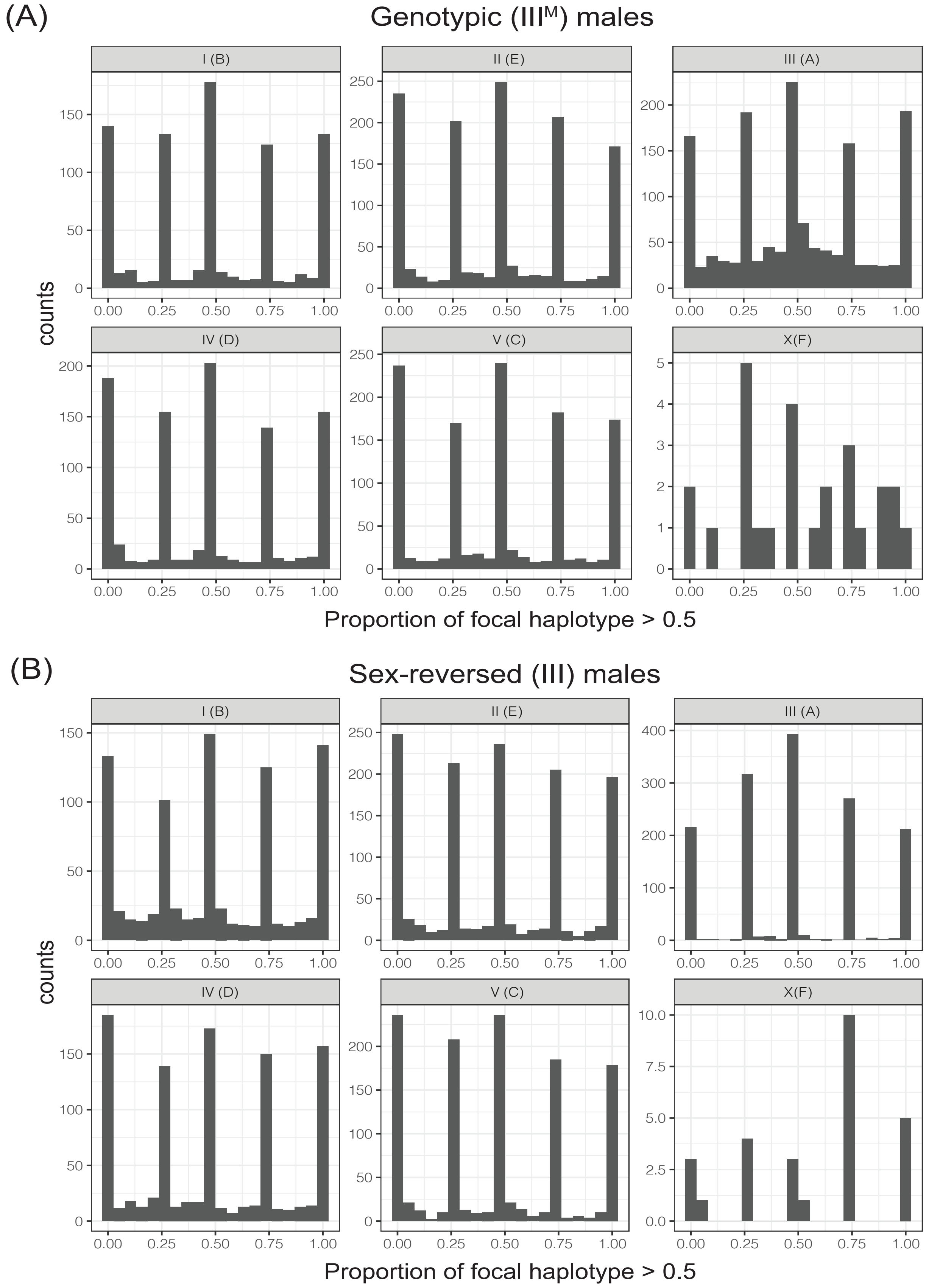
(A)
Genotypic (III^M) males
I (B)
II (E)
III (A)
IV (D)
V (C)
X(F)
counts
Proportion of focal haplotype > 0.5
(B)
Sex-reversed (III) males
I (B)
II (E)
III (A)
IV (D)
V (C)
X(F)
counts
Proportion of focal haplotype > 0.5

Supplementary Figure 3. Histograms of allele-specific expression (ASE) in genes for all chromosome are shown in the genotypic (III^M/III) males and the sex-reversed (III/III) males. Muller element nomenclature (from *Drosophila*) for each chromosome is given in parentheses (Meisel and Scott 2018). If a gene is expressed equally between two alleles, the proportion of the focal haplotype is 0.5; otherwise, the proportion is greater or less than 0.5.

Supplementary Table 1. Nine contigs in the III^M male assembly contain genes that are assigned to the third chromosome and have a %UFR>13.8. The genes on each contig are listed.

Contig ID	Gene	# copies of gene	%UFR of contig
ctg2522	LOC101894374	1	16.9
	LOC101894024	1	
	LOC101894537	1	
	LOC101894698	1	
ctg3539	LOC101888429	1	15.4
	LOC109612838	1	
ctg8407	LOC109613819	1	16.6
ctg1423	LOC101898200	6	14.5
ctg4658	LOC101898200	2	14.0
ctg5526	LOC101898200	1	17.3
ctg8144	LOC101898200	1	14.8
ctg9654	LOC101898200	1	20.1
ctg10432	LOC101898200	1	15.9

Supplementary Table 2. Genes on contigs with differentiated proto-Y (III^M) and proto-X (III) copies. There are two pairs of contigs (each pair contains a proto-Y contig and a proto-X contig). Results of a test for ASE in genotypic (III^M/III) males are shown in the second to last column and ASE of the sex-reversed (III^M/III) males in the last column. ASE was not estimated for some genes due to low sequence coverage.

			ASE category	
Contigs	Gene ID	Protein product	Genotypic (III^M/III) male	Sex-reversed (III/III) male
Proto-Y (III^M): ctg2382 (41,324 bps)<br>Proto-X (III): ctg1607 (66,106 bps)	LOC101893231	probable Xaa-Pro aminopeptidase 3	Moderate ASE	Extreme ASE
	LOC101892763	transcriptional adapter 1	Non-ASE	Extreme ASE
	LOC101893053	delta(3,5)-Delta(2,4)-dienoyl-CoA isomerase, mitochodiral	Not estimated	Not estimated
Proto-Y (III^M): ctg2522 (39,000 bps)<br>Proto-X (III): ctg1519 (56,029 bps)	LOC101894374	mitochondrial ornithine transporter 1-like	Not estimated	Not estimated
	LOC101894024	mitochondrial ornithine transporter 1	Moderate ASE	Extreme ASE
	LOC101894537	mitochondrial ornithine transporter 1-like	Extreme ASE	Moderate ASE
	LOC101894698	fast kinase domain-containing protein 5, mitochondrial	Extreme ASE	Moderate ASE

Supplementary Table 3. Missense alleles in coding sequences of two genes on the proto-Y and proto-X contigs. LOC101894698 has also five variable sites that are all synonymous, which are not shown in the table. Variants in coding sequences of the other three genes on the proto-Y and proto-X (LOC101892763, LOC101894024, and LOC101894537) are synonymous and not shown in the table.

Gene	Position at scaffold	III^M male genotype	III male genotype	III^M allele	Reference genome allele
LOC101894698	117354	T/A	A/A	T	T
	118649	C/T	T/T	C	C
	118909	T/C	C/C	T	T
LOC101893231	17382	C/T	C/C	T	C
	17429	A/G	A/A	G	A
	29021	G/A	A/A	G	G

Supplementary Table 4. Counts of ASE genes and non-ASE genes on each chromosome in genotypic (III^M/III) males and sex-reversed (III/III) males.

Chromosome (Muller element)	# genes with ASE in III^M males	# genes with non-ASE in III^M males	# genes with ASE in III males	# genes with non-ASE in III males	Odds ratio	95% CI of odds ratio
III(A)	456	413	438	412	1.038574	0.8555646 - 1.2606663
**genome except III(A)**	**1635**	**1089**	**1711**	**1010**	**0.886281**	**0.7933539 - 0.9900421**
I(B)	320	222	334	212	0.915002	0.7123784 - 1.1750187
II(E)	465	314	513	291	0.840134	0.6821523 - 1.0344483
IV(D)	394	249	395	215	0.861373	0.6798614 - 1.0908786
V(C)	448	296	460	288	0.947652	0.7654588 - 1.1730543
X(F)	8	8	9	4	0.720539	0.102255 - 4.754933

Supplementary Table 5. Counts of genes with ASE on each chromosome in genotypic (III^M/III) males and sex-reversed (III/III) males. The total number of genes (# genes) in each chromosome group with ASE measurements in both genotypic and sex-reversed males (second column), # genes with ASE in genotypic males and non-ASE in sex-reversed males (third column), and # genes with non-ASE in genotypic males and ASE in sex-reversed males (fourth column) are shown. Bold indicates statistical significance ($P < 0.05$)

		ASE in III^M males and non-ASE in III males	non-ASE in III^M males and ASE in III males	Fisher's Exact Test	
Chromosome (Muller element)	# genes	# genes	# genes	Odds ratio compared with III(A)	95% CI
III(A)	1420	95	76		
genome except III(A)	4201	**241**	**281**	**1.45665**	**1.014895 - 2.095777**
I(B)	824	51	59	1.444139	0.8688985 - 2.4078676
II(E)	1236	**68**	**86**	**1.578592**	**0.9961489 - 2.5100267**
IV(D)	966	**55**	**76**	**1.724145**	**1.063163 - 2.809137**
V(C)	1149	67	77	1.434875	0.8982881 - 2.2981210
X(F)	26	0	0	0	0 - Infinity

Supplementary Table 6. Counts of ASE genes based on the division of ASE measurements into five bins, following the rules described in the Methods. ASE proportions are sorted in the order of extreme (1st and 5th), moderate (2nd and 4th), and no (3rd) ASE. Only extreme ASE was used in the comparisons with non-ASE presented in the main text.

Chr (ME)	Sections for ASE proportions	# of genes in genotypic (III^M/III) Males			# of genes in sex-reversed (III/III) Males		
I(B)	1st (extreme ASE)	167	320	627	167	334	667
	5th (extreme ASE)	153			167		
	2nd (moderate ASE)	158	307		167	333	
	4th (moderate ASE)	149			166		
	3rd (non-ASE)	222	222	222	212	212	212
II(E)	1st (extreme ASE)	270	465	972	291	513	1013
	5th (extreme ASE)	195			222		
	2nd (moderate ASE)	251	507		258	500	
	4th (moderate ASE)	256			242		
	3rd (non-ASE)	314	314	314	291	291	291
III(A)	1st (extreme ASE)	215	456	1043	220	438	1050
	5th (extreme ASE)	241			218		
	2nd (moderate ASE)	312	587		333	612	
	4th (moderate ASE)	275			279		
	3rd (non-ASE)	413	413	413	412	412	412
IV(D)	1st (extreme ASE)	217	394	754	214	395	794
	5th (extreme ASE)	177			181		
	2nd (moderate ASE)	189	360		201	399	
	4th (moderate ASE)	171			198		
	3rd (non-ASE)	249	249	249	215	215	215
V(C)	1st (extreme ASE)	258	448	891	268	460	908
	5th (extreme ASE)	190			192		
	2nd (moderate ASE)	220	443		239	448	
	4th (moderate ASE)	223			209		
	3rd (non-ASE)	296	296	296	288	288	288
X(F)	1st (extreme ASE)	3	8	21	4	9	23
	5th (extreme ASE)	5			5		
	2nd (moderate ASE)	7	13		4	14	
	4th (moderate ASE)	6			10		
	3rd (non-ASE)	5	5	5	4	4	4

Supplementary Table 7. The number of annotated genes assigned to each chromosome (Muller elements in parentheses) and total length of the corresponding chromosomes in base pairs (bp).

Chromosomes (Muller elements)	# genes on chromosomes	Size in length (bp)
I(B)	2128	73015049
II(E)	3175	95424831
III(A)	2271	76855929
IV(D)	2400	76690558
V(C)	2660	81074832
X(F)	47	1486543
Unassigned	3859	345856202
Total	16540	750403944

Supplementary Data 1. A VCF file called with the RNA-seq reads
Supplementary Data 2. A VCF file called with the Oxford Nanopore reads
Supplementary Data 3. k-mer comparison in the III^M male genome assembly
Supplementary Data 4. IDP-ASE (allele-specific expression) output for the genotypic males
Supplementary Data 5. IDP-ASE (allele-specific expression) output for the sex-reversed males

Supplementary Data 1 and 2 are available at Dryad (https://doi.org/10.5061/dryad.280gb5mnk or https://datadryad.org/stash/share/6f4xn0dRAu8j2SQXCmqKICv95gYfpGzlhoR5SYLJZy0)

References

Carvalho AB, Clark AG. 2013. Efficient identification of y chromosome sequences in the human and drosophila genomes. Genome Res. 23(11):1894–1907.

Duronio RJ, Marzluff WF. 2017. Coordinating cell cycle-regulated histone gene expression through assembly and function of the Histone Locus Body. RNA Biol. 14(6):726–738.

Finnegan DJ. 2012. Retrotransposons. Curr Biol. 22(11):R432–R437.

McKay DJ, Klusza S, Penke TJR, Meers MP, Curry KP, McDaniel SL, Malek PY, Cooper SW, Tatomer DC, Lieb JD, et al. 2015. Interrogating the function of metazoan histones using engineered gene clusters. Dev Cell. 32(3):373–386.

Meisel RP, Scott JG. 2018. Using genomic data to study insecticide resistance in the house fly, Musca domestica. Pestic Biochem Physiol. 151:76–81.

Scott JG, Warren WC, Beukeboom LW, Bopp D, Clark AG, Giers SD, Hediger M, Jones AK, Kasai S, Leichter CA, et al. 2014. Genome of the house fly, Musca domestica L., a global vector of diseases with adaptations to a septic environment. Genome Biol. 15(10):466.

Sharma A, Heinze SD, Wu Y, Kohlbrenner T, Morilla I, Brunner C, Wimmer EA, van de Zande L, Robinson MD, Beukeboom LW, et al. 2017. Male sex in houseflies is determined by Mdmd, a paralog of the generic splice factor gene CWC22. Science (80-). 356(6338):642–645.

Volff JN. 2006. Turning junk into gold: Domestication of transposable elements and the creation of new genes in eukaryotes. BioEssays. 28(9):913–922.
